# Allergy immunotherapy with a hypoallergenic recombinant birch pollen allergen rBet v 1-FV in a randomized controlled trial

**DOI:** 10.1186/s13601-015-0071-x

**Published:** 2015-08-03

**Authors:** Ludger Klimek, Claus Bachert, Karl-Friedrich Lukat, Oliver Pfaar, Hanns Meyer, Annemie Narkus

**Affiliations:** Center for Rhinology and Allergology, An den Quellen 10, D-65183 Wiesbaden, Germany; Upper Airway Research Laboratory, University Hospital Ghent, Ghent, Belgium; Institut für Atemwegsforschung (IFA) GmbH, Düsseldorf, Germany; Medical Department, Allergopharma GmbH & Co. KG, Reinbek, Germany

**Keywords:** Allergy immunotherapy, Subcutaneous immunotherapy, Birch pollen, Allergic rhinitis, Rhino-conjunctivitis, Birch pollen, Recombinant Bet v 1, Hypoallergenic variant, Folding variant, Recombinant allergen

## Abstract

**Background:**

Pollen extracts and chemically modified allergoids are used successfully in allergen immunotherapy (AIT). Recombinant extracts offer potential advantages with respect to pharmaceutical quality, standardization and dosing. A hypoallergenic recombinant folding variant of the major birch pollen allergen (rBet v 1-FV) was compared with an established native birch preparation. A pre-seasonal, randomized, actively controlled phase II study was performed in birch pollen allergic rhino-conjunctivitis with or without asthma, GINA I/ II. 51 patients (24 rBet v 1-FV, 27 native extract) started therapy with subcutaneous allergen immunotherapy (SCIT). Primary end-point was a combined symptom medication score (SMS), changes in nasal provocation test, visual rating score and specific antibody responses secondary end-points.

**Findings:**

After one pre-seasonal treatment course the combined SMS was 5.86 (median; IQR: 14.02) for the rBet v 1-FV group versus 12.40 (median; IQR: 9.32) for the comparator during the three weeks pollen season (*p* = 0.330). After treatment in the second year, scores were 3.00 (median; IQR: 6.50) and 2.93 (4.86) respectively. Allergen tolerance in a nasal provocation test improved to a comparable extent in both groups. Significant increases in birch pollen-specific IgG1 and IgG4 were observed in both treatment groups following the first treatment phase and remained significantly raised until the end of the study.

**Conclusion:**

In this first in man, proof of concept phase II trial no statistical difference between rBet v 1-FV and an established natural pollen extract could be observed. rBet v 1-FV could be administered in higher doses than the native protein with no increase in adverse effects.

**Trial registration:**

The study was registered in clinicalTrials.gov (NCT00266526).

## Introduction

Subcutaneous immunotherapy (SCIT) has been shown to be clinically efficacious in numerous controlled clinical studies and is the only curative approach towards allergy treatment recommended in a WHO Position Paper [1].

Recombinant preparations are an ideal basis for development of diagnostic and therapeutic preparations, since they are molecularly defined and can be produced in high purity with consistent quality, thereby circumventing many of the difficulties associated with natural allergen extracts and their standardization.

In order to reduce the risk of IgE-mediated therapy-induced side effects a folding variant of recombinant Bet v 1 (rBet v 1-FV), the major birch pollen allergen, has been developed [2].

This is the first report of a randomized, controlled, proof of concept study comparing two pre-seasonal treatment courses of SCIT with either rBet v 1-FV or an approved native birch pollen depot extract. The objective of the study was to determine the safety and efficacy of the recombinant preparation, and to investigate whether treatment with a single major allergen as opposed to a whole extract can be clinically effective.

The publication is performed according to CONSORT guidelines.

## Materials and methods

The study was performed in accordance with the Guidelines for Good Clinical Practice [3] and approval of local ethics committees.

Treatment was conducted between October and March in two consecutive years. Dosage was increased progressively with 8 injections of aluminum hydroxide adsorbed rBet v 1-FV [4] (5 μg/mL Strength A, 100 μg/mL Strength B) at 7-day intervals, cumulative dose 157.5 μg; comparator: 14 injections, 3 strengths (50, 500 and 5,000 (therapeutic units) TU/mL), 16,325 TU, and continued until the onset of the birch pollen seasons.

**Fig. 1:**
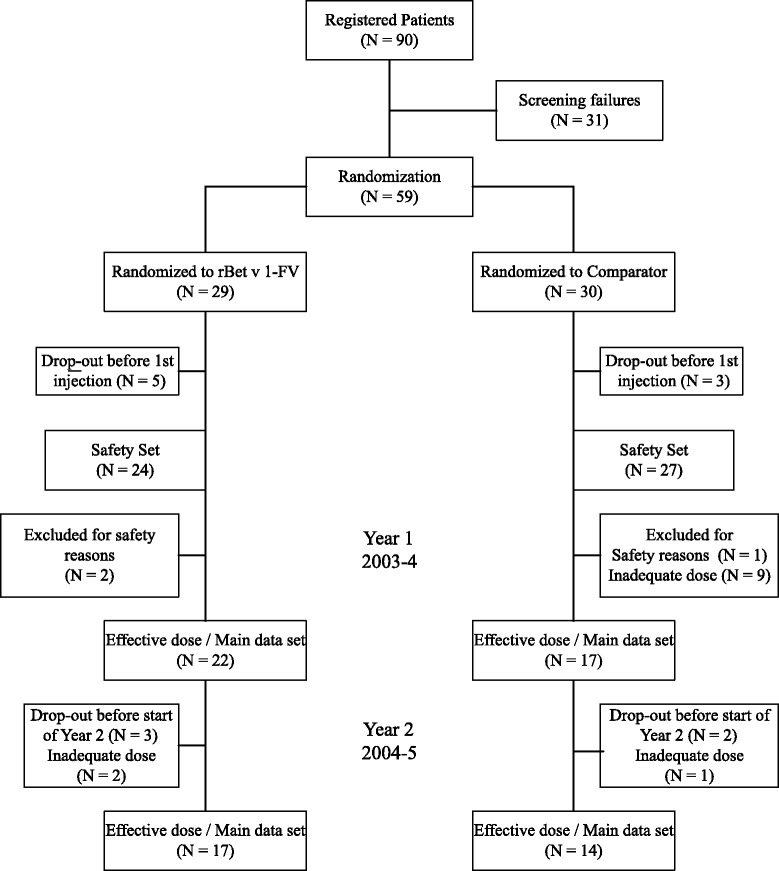
Flow chart documenting progress through the study of those patients included in the main data sets

Patients with birch pollen rhino-conjunctivitis, with or without asthma (GINA 1 or 2 [5]), requiring medication during the previous pollen season, with positive skin prick test for birch, specific IgE-RAST ≥ 2 (ImmunoCAP®) and a positive nasal provocation test (NPT) [6], fulfilling usual AIT exclusion criteria, were recruited (Fig. 1).

Determination of outcome measures required that subjects achieved an adequate dose, defined as either at least one injection of the maintenance dose of rBet v 1-FV (strength B/0.8 mL, 80 μg) or 13 injections of the comparator (Novo-Helisen® Depot birch pollen, Allergopharma GmbH & Co. KG) with at least one dose of 2,000 TU.

Main data set in the first year: 39 subjects (rBet v 1-FV 22, comparator 17), second year: 17 and 14 of those subjects respectively. Demographic data is shown in Table 1.

**Table 1:** Demographic and baseline data

		rBet v 1-FV	Comparator birch pollen depot preparation	Reference group
		*n* = 24	*n* = 27	*n* = 34
Gender (n)	M/F	10/14	13/14	10/24
Age (years)	Mean ± SD	41.00 ± 12.28	37.70 ± 9.83	38.38 ± 11.58
	Range	20–63	22–60	23–59
Bet v 1 specific IgE at recruitment out of season (kUA/L)	Mean ± SD	21.79 ± 24.96	21.28 ± 39.76	n.a.
	Range	0.6–101.2	0.7–168.1	
Allergic symptoms				
Conjunctivitis	n (%)	24 (100 %)	27 (100 %)	34 (100 %)
Rhinitis	n (%)	24 (100 %)	26 (96 %)	34 (100 %)
Cough/sibilant rhonchi	n (%)	6 (25 %)	3 (11 %)	20 (59 %)
Allergic asthma	n (%)	9 (38 %)	11 (41 %)	15 (44 %)
Atopic dermatitis	n (%)	1 (4 %)	0 (0 %)	4 (12 %)
Duration of symptoms (years)	Mean ± SD	11.75 ± 10.86	12.52 ± 9.34	14.09 ± 6.45
	Range	1–43	1–33	2–26
Median SMS on 15th March first treatment year^a^		3.0	3.0	5.5

n.a.: not available; ^a^first day on which SMS data was collected

Adverse events were coded according to the Medical Dictionary for Regulatory Activities (MedDRA).

Symptom Medication Score (SMS) in the birch pollen seasons was the primary outcome measure for efficacy, using a validated score [7]. Subjects had access to short-acting, non-prophylactic symptomatic medication (short-acting topical antihistamine as first-line treatment, oral antihistamine for more severe symptoms; short-acting bronchodilator for asthma and inhaled steroids scored only if dose was changed).

Final evaluation was based on a 21-day period of main birch pollen exposure (median area under the curve (AUC), 7 days before until 13 days after peak pollen count).

Nasal provocation test was performed at inclusion and prior to the birch pollen seasons according German guideline [6].

### Immunoglobulin measurements

Birch pollen, Bet v 1, 2 and 4 specific IgE was measured at inclusion (ImmunoCAP®), birch pollen specific IgG1, IgG4 and IgE responses by ELISA [8]: 1, screening before SCIT; 2, after up-dosing first season; 3, after first pollen season; 4, after up-dosing second season; and 5, after second pollen season.

Pollen counts were provided by the European Aeroallergen Network (https://ean.polleninfo.eu/Ean/, (siegfried.jaeger@meduniwien.ac.at).

### Reference group

34 subjects participating in the baseline year of a separate birch pollen SCIT study being conducted under identical conditions in the same geographical region of Germany.

### Statistical analysis

The AUC for the treatment groups were compared using confidence intervals to draw conclusions on possible superiority, non-inferiority or equivalence of efficacy. The two-sided Wilcoxon-Mann–Whitney U-Test at α = 0.05 was applied in the analysis of antibody responses using SPSS Version 14.02 (SPSS Inc., Chicago, USA).

## Findings

### Application of study medication

265 rBet v 1-FV injections were administered during the first treatment year, 228 during 2nd year (median 11 and 12 per patient); comparator 349 and 353 (median 14 and 18). The maximum dose of 80 μg rBet v 1-FV was achieved in 22/24 subjects in the 1st year, one achieved 20 μg, one 4 μg (2nd year: 17 (80 μg), 2 (2 μg)). Only 7/27 subjects achieved the recommended dose of 5,000 TU with the comparator, equivalent to 16 μg natural Bet v 1, 4/27 achieving 4,000 TU, 7/27 3,000 TU, 4/27 2,000 TU and 5/27 1,000 TU or less (2nd year: 18 (5,000 TU), 3 (4,000/3,000/40 TU).

### Symptom medication score (SMS)

**Fig. 2:**
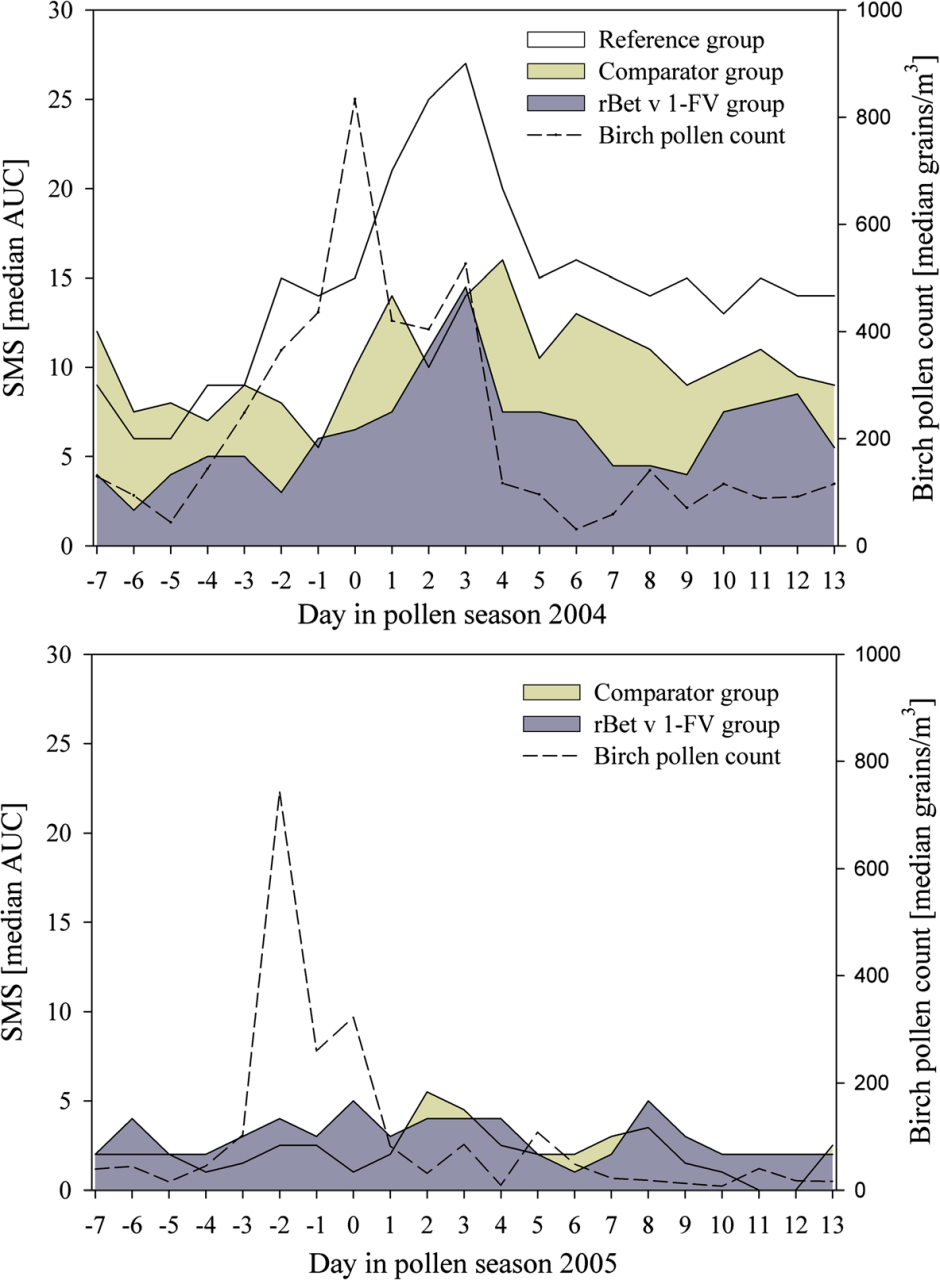
Course of daily median SMS and median pollen counts during the 21-day observational periods in the birch pollen season after one (above) and two courses (below) of SCIT, respectively

With a peak pollen count of 834 grains/m^3^ during the first pollen season the median AUC of SMS for the rBet v 1-FV group was 5.86 (IQR: 14.02), substantially lower than the 12.40 (IQR: 9.32) recorded for the comparator group (*p* = 0.330), reference group 14.67 (IQR: 13.64) (Table 2 & Fig. 2).

**Table 2:** Median AUC of the symptom scores, medication scores and combined SMS for the 21 day observation period during main birch pollen exposure in each of the two seasons

	rBet v 1-FV	Comparator	Wilcoxon–Mann–Whitney U-Test
	median (IQR)	median (IQR)	*p*-values
First treatment year			
Symptom score	3.10 (9.36)	5.69 (4.71)	0.400
Medication score	1.43 (3.10)	2.83 (5.75)	0.285
SMS	5.86 (14.02)	12.40 (9.32)	0.330
Second treatment year			
Symptom score	2.81 (4.02)	1.67 (2.41)	0.393
Medication score	0.29 (1.36)	1.05 (3.50)	0.730
SMS	3.00 (6.50)	2.93 (4.86)	0.812

Substantial cross-reacting tree pollen counts occurred during the few weeks prior to the 21 day observation period, and probably accounted for the differences in SMS at the beginning of the 21 day observation period (7th April) (Fig. 2). The median SMS values for the three groups on 15th March, the first day on which data was collected, were 3.0, 3.0 and 5.5 respectively.

In the second year (peak pollen count 827 grains/m^3^) the median SMS for the rBet v 1-FV and comparator groups were 3.00 (IQR: 6.50) and 2.93 (IQR: 4.86) respectively (Table 2).

### Specific antibody responses

**Fig. 3:**
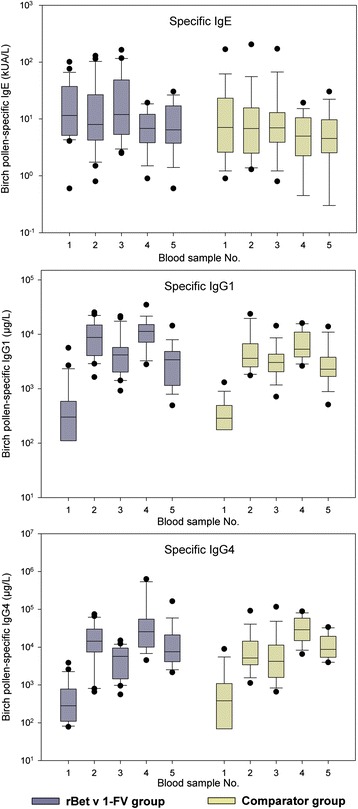
Birch pollen-specific IgE, IgG1 and IgG4 concentrations. Median values with 25th/75th and 10th/90th percentiles represented by boxes and error bars respectively, outliers by points. Time points: 1, screening before SCIT; 2, after up-dosing first year; 3, after pollen season first year; 4, after up-dosing second year; and 5, after pollen season second year

Baseline birch pollen-specific IgE levels as well as levels during treatment were similar for rBet v 1-FV and the comparator groups; IgG1 and IgG4 levels were comparable at baseline, showed significant increases with marginally stronger responses in the rBet v 1-FV group (Fig. 3) and further increases in the second year.

### Nasal provocation test

At entry there were no significant differences between the two groups in NPT. An enhanced tolerance threshold, with at least a three-fold increase in the tolerated allergen concentration, was seen in 8/15 subjects in the rBet v 1-FV group (8/14 comparator) after SCIT in the first year. In the second year enhanced tolerance was seen in 11/12 rBet v 1-FV (1 subject showing no change), as compared with only 6/11 in the comparator group (3 subjects no change, 2 deterioration (Table 3)).

**Table 3:** Nasal provocation test response thresholds

A								
rBet v 1-FV		Threshold response after treatment in 1st year						
	BU/mL	555	1,666	5,000	15,000	45,000	150,000	negative
Threshold response at baseline	555	**3**	3	0	0	0	0	1
	1,666	1	**2**	1	2	0	0	0
	5,000	0	1	**0**	1	0	0	0
	15,000	0	0	0	**0**	0	0	0
	45,000	0	0	0	0	**0**	0	0
rBet v 1-FV		Threshold response after treatment in 2nd year						
	BU/mL	555	1,666	5,000	15,000	45,000	150,000	negative
Threshold response at baseline	555	**0**	4	0	2	0	0	0
	1,666	0	**0**	2	0	1	1	0
	5,000	0	0	**1**	0	0	1	0
	15,000	0	0	0	**0**	0	0	0
	45,000	0	0	0	0	**0**	0	0
B								
Comparator		Threshold response after treatment in 1st year						
	BU/mL	555	1,666	5,000	15,000	45,000	150,000	negative
Threshold response at baseline	555	**0**	0	2	0	0	0	0
	1,666	2	**2**	3	1	0	0	0
	5,000	0	1	**0**	0	2	0	0
	15,000	0	0	0	**0**	0	0	0
	45,000	0	0	0	1	**0**	0	0
Comparator		Threshold response after treatment in 2nd year						
	BU/mL	555	1,666	5,000	15,000	45,000	150,000	negative
Threshold response at baseline	555	**1**	0	0	0	0	0	0
	1,666	1	**1**	4	0	0	0	0
	5,000	0	0	**1**	0	1	0	1
	15,000	0	0	0	**0**	0	0	0
	45,000	0	0	0	1	**0**	0	0

Data indicate numbers of subjects and their responses at baseline compared with that after 1 year or 2 years of treatment with (A) rBet v 1-FV or (B) comparator

Bold printed numbers represent no change in response, and data to the right or the left an increase or decrease in tolerance respectively

### Adverse events

During the two pre-seasonal treatment courses at least one local or systemic reaction with at least possible relationship to trial medication was recorded in 22/24 (91.7 %) of the rBet v 1-FV treated subjects and 23/27 (85.2 %) of comparator; systemic reactions in 42 % of rBet v 1-FV, 41 % comparator.

Two serious adverse events occurred during the study, both in the same patient on the same day 5 weeks after administration of the last rBet v 1-FV injection (bursitis, acute exacerbation right shoulder and trigger-thumb right) being assessed as not treatment related.

All reactions (Table 4) are grouped according MedDRA system organ classes.

**Table 4:** Adverse events reported with a least a possible relationship to SCIT for both study groups, classified in accordance with MedDRA

System organ class	AEs reported (Preferred terms)	rBet v 1-FV (*n* = 24)		Comparator (*n* = 27)	
		No. of patients with at least one AE (%)	No. of Events	No. of patients with at least one AE (%)	No. of Events
Cardiac disorders	Cardiovascular disorder	1 (4)	1	0 (0)	0
Eye disorders	Eye pruritus, Lacrimation increased, Conjunctivitis	2 (8)	3	3 (11)	3
Gastrointestinal disorders	Oral pruritus, Nausea	1 (4)	1	1 (4)	1
General disorders and administration site conditions	Fatigue, Injection site erythema, Injection site swelling, Injection site pruritus, Injection site induration, Injection site pain, Injection site warmth	16 (67)	101	20 (74)	151
Immune system disorders	Eyelid oedema, CSF monocyte count, Conjunctivitis allergic, Rhinitis allergic, Urticaria, Allergic cough, Asthma	7 (29)	23	5 (19)	11
Infections and infestations	Herpes simplex, Rhinitis	2 (8)	3	2 (7)	2
Investigations	Peak expiratory flow rate decreased	1 (4)	1	1 (4)	2
Psychiatric disorders	Nervousness	0 (0)	0	1 (4)	1
Respiratory, thoracic and mediastinal disorders	Asthma, Nasal congestion, Nasal discomfort, Pharyngolaryngeal pain, Chest discomfort, Cough, Dyspnoea	3 (12)	5	7 (26)	12
Skin and subcutaneous tissue disorders	Erythema, Exanthem, Neurodermatitis, Rash pruritic, Swelling face, Urticaria, Urticaria generalised	6 (25)	8	1 (4)	1

## Discussion

This is the first in man clinical study using a hypoallergenic recombinant folding-variant of Bet v 1 for the treatment of seasonal allergy. The study was designed as a randomized, controlled trial to determine potential clinical efficacy by open comparison (because of different up-dosing schemes) with an established birch pollen preparation.

The median daily SMS for the rBet v 1-FV group was substantially less than that for the birch pollen extract treated group 5.86 (median; IQR: 14.02) as compared with 12.40 (median; IQR: 9.32) after the first pre-seasonal treatment. This difference equates to the daily use of one tablet anti-histamine. During the 2nd pollen season the SMS for the two groups were very comparable, indicating that the whole pollen extract was able to achieve the same effect as the recombinant molecule, but took longer to do so. The difference in SMS was not statistically significant, but it should be noted that this was a first in man study with a totally new product and because of safety reasons not powered to demonstrate differences between treatments. However, the lack of power calculation and the absence of a placebo group is an important limitation as it prevents any direct assessment of the significance of the clinical improvement. The comparison with a reference group from the same geographical region from the baseline season of a phase III trial with rBet v 1-FV (NCT00309062) helps to put the data into perspective.

The enhanced tolerance threshold compared with baseline in NPT after the 2nd treatment course was more pronounced in rBet v 1-FV (11/12 subjects) compared to NHD (6/11). Single pre-seasonal treatments with either hypoallergenic fragments or a trimeric form of recombinant Bet v 1 showed increased tolerance within the groups, but not in comparison to placebo [9]. The advantage in favor of the recombinant preparation in the present study may reflect the higher therapeutic dose and/or its superior efficacy.

There were no significant changes in birch pollen or Bet v 1 specific IgE in either group, although a slight downward trend was apparent in the 2nd year of the study. Such decreases have been seen with longer treatment protocols including recombinant grass pollen allergens after 20 months of therapy [10], and pre-seasonal treatment with a grass pollen allergoid over 2 years [11]. These results suggest that AIT can cause at least partial suppression of those Th2 cytokines essential for IgE-production.

The large and significant increases of birch pollen specific IgG4 levels at maximum dosing, indicate that rBet v 1-FV has a strong immunogenic effect, slightly in excess of that of the allergen extract; possibly explained by the random coil structure facilitating processing by antigen presenting cells [12].

The rBet v 1-FV was well tolerated and the safety profile was comparable with that of the natural pollen preparation. A permanent dose-reduction was not necessary in subjects with systemic reactions, and the maintenance dose of 80 μg protein could be realized in nearly all subjects. The comparable safety data with the two preparations in the present study, despite the five-fold higher major allergen dose of the recombinant preparation, indicates a possible advantage for the hypoallergenic rBet v 1-FV.

This first in man proof of concept clinical study of SCIT using a folding-variant of an rBet v 1- vaccine for the treatment of seasonal hay fever has demonstrated good clinical tolerance and efficacy after just one pre-seasonal course comparable to a native birch pollen preparation. The induction of strong allergen-specific IgG antibody responses demonstrates the immunogenicity of the rBet v 1-FV and confirms that the hypoallergenic characteristics are not detrimental to its immune-modulatory potential. Subsequent clinical DBPC-trials are warranted to confirm its clinical efficacy.
